# GWEHS: A Genome-Wide Effect Sizes and Heritability Screener

**DOI:** 10.3390/genes10080558

**Published:** 2019-07-24

**Authors:** Eugenio López-Cortegano, Armando Caballero

**Affiliations:** Departamento de Bioquímica, Genética e Inmunología, Universidade de Vigo, 36310 Vigo, Spain

**Keywords:** GWAS, missing heritability, prediction of complex traits, big data

## Abstract

During the last decade, there has been a huge development of Genome-Wide Association Studies (GWAS), and thousands of loci associated to complex traits have been detected. These efforts have led to the creation of public databases of GWAS results, making a huge source of information available on the genetic background of many diverse traits. Here we present GWEHS (Genome-Wide Effect size and Heritability Screener), an open-source online application to screen loci associated to human complex traits and diseases from the NHGRI-EBI GWAS Catalog. This application provides a way to explore the distribution of effect sizes of loci affecting these traits, as well as their contribution to heritability. Furthermore, it allows for making predictions on the change in the expected mean effect size, as well as in the heritability as new loci are found. The application enables inferences on whether the additive contribution of loci expected to be discovered in the future will be able to explain the estimates of familial heritability for the different traits. We illustrate the use of this tool, compare some of the results obtained with those from a previous meta-analysis, and discuss its uses and limitations.

## 1. Introduction

Genome-Wide Association Studies (GWAS) have become the standard tool to find new loci underlying the genetic basis of many complex traits and diseases [1], yet the nature of their genetic architecture, i.e., the number and relative contribution of loci to genetic variation, is mostly unknown [2,3]. In fact, though thousands of SNPs (single nucleotide polymorphisms) have been found for many human traits, most of their genetic variance remains unexplained (i.e., the missing heritability problem) [4,5]. It is generally accepted that this missing heritability is mainly due to the additive contribution of hundreds to thousands of loci of small effect size that are not detected by current GWAS because of a lack of statistical power [6,7]. However, new SNP discoveries tend to have lower and lower effect sizes contributing progressively less to heritability [8,9]. Furthermore, it has been suggested that familial estimates of narrow-sense heritability could be overestimated because of environmental and non-additive genetic components of variation [10], as well as different sources of genotype-covariance factors [4,11,12,13]. Thus, it is possible that the heritability explained by GWAS detected variants may not be ever able to explain familial heritability estimates, at least for some traits [9].

Efforts to find all variants responsible for human traits have led to the creation of big consortiums [7,14] as well as public databases of GWAS results, such as the NHGRI-EBI GWAS Catalog [15,16] or the UK Biobank [17]. These constitute a great source of information for researching the genetic basis and architecture of complex traits, and many software packages and tools are being published to make the most of these resources. For example, the GeneATLAS [18] provides an easy way to query information related to traits and gene variants from the UK Biobank, including statistics as their effect sizes or significance. Regarding the GWAS Catalog, the R package ‘gwascat’ [19] also allows GWAS results to be queried from an R environment. Other tools have also been developed with regard to these databases, such as CPAG (Cross-Phenotype Analysis of GWAS) to investigate pleiotropy [20]. However, as far as we know, there are no applications using information from the GWAS Catalog that estimate heritability from GWAS results or that make inferences on the expected heritability as new candidate variants are found.

Though not very extended, the number of open-source tools is also increasing in the last years, and this is also useful for the scientific community, who is becoming more aware of the problems derived from the lack of reproducibility in science [21,22,23]. However, these tools are usually more focused on methods to be run than on communicating the output results (e.g., by providing a system to visualize them). In this regard, statistic languages as R [24] and plain text file formats based on markdown have become increasingly popular for building research reports [25], as they give a reproducible and convenient way to share information, including statistical results that can react interactively depending on the recipient’s input, enhancing their communicative potential. Here we present GWEHS (Genome-Wide Effect size and Heritability Screener), an application and online resource for exploring the distribution of locus effect sizes and their contribution to heritability using the GWAS Catalog database. Inferences on the change in the mean effect size and expected heritability to be found with an increasing number of loci discoveries are also performed by using regression analyses as well as by sampling loci from an expected distribution of effect sizes. Overall, the aim of this tool is to provide an easy access to the information stored at the GWAS Catalog, as well as to explore and give an overview of the genetic architecture of human complex traits, following methods developed by López-Cortegano and Caballero [9]. This application is distributed as an open-source R markdown file, and accessible online without needing to install any software or download the raw GWAS Catalog database.

## 2. Methods

The application presented here, relies on the SNP information published in hundreds of scientific publications, and that is made publicly accessible thanks to the NHGRI-EBI GWAS Catalog [15,16]. In a first step, the GWAS Catalog is downloaded and pre-processed to retain only the most significant and robust information. These tasks are performed periodically following an R script and then deployed together with the application to a hosting server. In a second step, an interactive R markdown file (i.e., the application) is used to perform analyses based on the user’s input. Most of the scripting was done in R language v.3.6 [24], and the interactive application is based on R markdown v.1.13 [26] and the Shiny package v.1.3.2 [27]. These have been submitted to a long-term repository at GitLab (https://gitlab.com/elcortegano/GWEHS) [28]. The online application is currently accessible at http://gwehs.uvigo.es. Its status will always be indicated at the GitLab repository, together with all documentation files. The repository also contains instructions to run the server locally both using R or a Docker image. Below we briefly summarize the details of these scripts.

### 2.1. Data Processing

In this section we describe methods used to process the GWAS Catalog data. These steps are run on the server side before the deployment of the application by means of a ‘preprocessing.sh’ script (see the repository link above), and thus do not take part on the analyses run when using GWEHS. A friendly summary of the data processing can be found at the repository’s wiki page (https://gitlab.com/elcortegano/GWEHS/wikis/home) [29].

#### 2.1.1. Data Acquisition and Processing the GWAS Catalog

The GWAS Catalog database is publicly available for download at https://www.ebi.ac.uk/gwas/ [30]. For the analyses and results shown here, we refer to the GWAS Catalog v1.0.2 (e96 r2019-06-20) accessed on June 30, 2019. From the raw GWAS Catalog, we only retained information on the SNPs identity, mapped gene, effect size, risk allele frequency, *P*-value (*P*), genomic position (chromosome number and position in base pairs), PubmedID (PMID), publication date, population sample information, and the disease or trait examined. In order to avoid ambiguity, we will refer here to the mapped gene by the term “locus”, as they sometimes include inter-genic regions (identified by a hyphen character in the gene name). However, the application will allow for more flexible definitions, as loci can also be filtered by setting a physical distance in mega bases (e.g., 1 Mb; see below). Loci names are truncated to the first one when there are several names separated by comma (due to overlapping transcripts). These names were usually the same, and coincident with the reported gene name as shown by the GWAS Catalog. All SNP entries with incomplete information, or with a –log(*P*) lower than 5 (i.e., *P*-value higher than 10^−5^) are removed. Population sample information is given in text form by means of two different fields: ‘Initial sample description’ and ‘Replication sample description’. From that, we estimate the population sample size as the sum of the sample’s sizes extracted from both descriptions.

After the first filtering step, all traits are examined for their associated number of loci and PMIDs. Traits with a number of loci lower than 30 are also excluded, as a minimum number of loci is desired to fit their effects to a distribution (see Section 2.2.1 below). Traits represented by a low number of PMIDs (less than 3) are also excluded, as at least three points are needed to fit an appropriate regression model. These two filtering criteria by number of loci and PMID are called one after another recursively until both conditions are satisfied. The remaining traits and SNPs are then classified by their effect type as explained below, before saving a local database of the processed catalog. This is run together with an additional filtering function that groups loci by a unique combination of trait and PMID identifiers (e.g., loci of trait A described in PMID 1 are grouped separately from loci of trait A in PMID 2 and from loci of trait B in PMID 1). We remove those groups whose SNPs contribute together more than 1.0 to heritability, which may occur, for example, if the submitted effect size is not in phenotypic standard deviation units. This latter filtering step is only done for traits with effects measured as beta-coefficient, since for effects measured as odd ratio the contributions to heritability depend on a prevalence value and thus can change widely (see Section 2.1.2 below).

#### 2.1.2. Classifying Locus Effect Sizes and Inferring Beta-Coefficients from Odd Ratios

Traits with loci effects measured as beta-coefficient (BETA) or odd ratios (OR) are treated separately. From the GWAS Catalog, it is not always clear when an effect belongs to one type or the other. As a proxy, we avoid ambiguity by regarding the minimum effect size by PMID and trait, and classifying the corresponding locus effect as OR if this value is equal to or higher than 1 (the minimum value), or BETA otherwise. For a few traits there is such a conflict as different PMIDs are classified with discordant effect types. For these traits, we selected the most representative effect type (i.e., the one with more SNP entries), and removed the other one. In any case, the effect type used will always be indicated in the box located together with the plot of distribution effect sizes (see examples below). At this point, the preprocessing is ended for all traits with BETA effects. Since these effects are measured in phenotypic standard deviations, their contribution to additive genetic variance, *V_A_*, equals the narrow-sense heritability (i.e., *V_A_ = h*^2^).

Regarding OR traits, their effects are transformed to BETA effects using the method developed by So et al. [31]. In short, this method takes the SNP’s OR value, the risk allele frequency and a value of prevalence, and computes the SNP contribution to heritability (i.e., variance in liability) considering the differences in mean liability for each genotype. From the genotype frequencies (*P*) and disease prevalence (*k*), penetrance (*f*) can be calculated, assuming an additive effect of the risk ratios (*RR*) (i.e., the risk ratio in the heterozygote is half that in the homozygote for the risk allele), following the expression *f*_AA_ = *k* / (*P_AA_* + ½*P_Aa_RR* + *P_aa_RR*), where the suffix ‘*A*’ refers to the wild-type allele, and lower-case ‘a’ to the risk allele. Risk ratios can be computed by means of the OR value and penetrance simply as *RR = OR* / (1 *+ f_AA_* (*OR* – 1)). These two expressions must be solved recursively until convergence. Then, from the values of penetrance and risk ratios, the liability mean is computed for each genotype (assuming Hardy-Weinberg equilibrium), and the corresponding variance in liability (the SNP-contributed *h*^2^) is obtained. This SNP-contributed *h*^2^ can be equaled to the classical expression 2*α*^2^q(1 − q) [32] (*q* being the risk allele frequency), from which the effect size in phenotypic standard deviations (*α*, which is equivalent to BETA) is obtained. Note that OR traits need a prevalence value, which will affect the magnitude of the effect size in phenotypic standard deviation units. We advise to insert the prevalence value as accurate as possible, although it has been reported that deviations from the true value of prevalence, when this is small, have little impact on this transformation process [33]. For OR traits, possible values of effect size are pre-computed for a wide range of prevalence values and saved in a database that is read at execution time. The prevalence values considered ranged from 0 to 50% in steps of 10^−4^. Note that effect sizes (*α*) are estimates of the average effect of allelic substitution [32] and, therefore, take into account possible non-additive (dominance and epistatic) effects of the loci [9], but neither dominance nor epistatic variance components can be computed, so broad-sense heritability estimates cannot be estimated. For the analyses performed here, we used penetrance values reviewed by López-Cortegano and Caballero [9].

### 2.2. Analyses on Loci Effect Size and Heritability

The application has an exploratory tool and an inference tool. These are explained in the two subsections below. In general, the methodology followed is that used by López-Cortegano and Caballero [9], though it has been simplified at some steps to facilitate its automation as well as performance. For example, the automatic clustering of traits based on genetic similarity followed by López-Cortegano and Caballero [9] cannot be made by GWEHS and it is left to be made manually by the user. A full list of all available traits can be downloaded from the application, including their classification by effect type. Other options, however, have been extended. For example, the PMIDs can be sorted for analysis during inference of mean effect sizes and trait heritability, not only by population size *N* (the default option), but also by publication date, which is more easily curated and easy to read from the Catalog. Nevertheless, the most recent analyses are usually those with the highest sample sizes, so it is frequent that a consideration of studies by publication time or sample size are coincident, serving as an indicator of the statistic power and/or the power of the methods used in GWAS.

Depending on the user’s input, one or more biological traits are selected and filtered regarding their *P*-value and/or physical distance between SNPs. If more than one trait is selected, they are analyzed as a whole, with the restriction that traits classified with different effect types (BETA and OR) cannot be mixed. When loci are filtered on the basis of their genomic position, a sliding window of custom size is defined so that only one locus will remain in it, that with the most significant associated SNP. A filtering can also be made by removing outliers from the data using Tukey’s classical method [34]. After these filtering steps based on the user’s input, the traits are evaluated again for a minimum number of loci (30) and PMIDs (3), so it may occur that if the filtering (*P*-value, genomic window or removal of outliers) is too restricted, the analysis cannot be done. The application will also inform on any errors that could arise from the used options in the screen corresponding to the distribution of effect sizes. All the methods described in this section are run from the main ‘gwehs.Rmd’ file (made available in the repository link above and also readable by clicking the ‘Source code’ button in the application) at execution time.

#### 2.2.1. Effect Sizes Distribution, and Contribution to Heritability

Exploratory analyses are based on a set of unique loci which putatively constitute the genetic basis of the trait found so far in all available to date GWAS. For the dataset used here, only the SNP found in the PMID with the highest value of *N* is retained per locus. If two or more SNPs associated with a given locus share the same value of *N* (or publication date if this is selected as sorting criterion), that with the lowest *P*-value is kept. Thus, a given locus (GWAS named gene or intergenic sequence) is assigned the estimated effect and frequency of that particular SNP. The loci effects are fitted into a set of known parametric distributions (beta, exponential, gamma, gaussian, logistic and log-normal) using the R package ‘fitdistrplus’ [35] and a maximum likelihood estimation method. The best fit is selected by using the Akaike information criterion (AIC) [36], though the distribution used can also be modified by the user. The contributions of these loci to heritability are also given based on an arbitrary classification in three classes: low, medium and high effect sizes, where the percentage of loci belonging to each of these is determined by the user (to a maximum percentage of 50% for a class).

#### 2.2.2. Inferring the Mean Effect Size and Missing Heritability

The inference analyses are based on a dataset where loci are sorted by population sample size *N* as before, but now in a cumulative way, so that loci from studies with higher *N* (i.e., statistic power) are added to those found in studies with lower *N*. To make the inference process dependent only on the discovery of new variants and not on the quality of the estimates at different PMIDs, the effects and frequencies of loci repeated between PMIDs are updated with those of the PMID with the highest *N*. For example, if locus L has an effect *α* and frequency *q* in the PMID with the highest *N*, then all entries of L in PMIDs with lower *N* will be assigned the same effect *α* and *q*, independently of the values present in the Catalog, as it is assumed that the estimation with the highest *N* is the most accurate one. If, alternatively, sorting is made by publication date rather than population sample size, the same procedure is followed but based on the criterion of date.

Three different regression models are considered in order to fit the change of mean loci effect size and trait heritability to the loci found with an increasing number of PMIDs. By default, a two-parameter exponential regression (*Y = aX^b^*) is used, but linear (*Y = a* + *Xb*) and four-parameter logistic regression *Y* = *c* + (*d* − *c*) / (1 + exp(b*(*ln*X* − ln*e*))) models are also available, with *Y* and *X* being the dependent and independent variables respectively (here the mean effect size or heritability, and the number of loci, respectively), and letters from ‘*a*’ to ‘*e*’ the regression parameters to be estimated. In all cases, their corresponding values of AIC are returned. We choose the exponential regression as default based on a previous meta-analyses where it gave the best fit [9], but note that the fit depends on two starting parameter values and convergence may not occur in some circumstances, so it is advised to check these AIC values and change the regression method used if necessary. Note also that at least 3 PMIDs will be required per trait in order to perform a regression analysis.

Inferences on heritability are done by means of two different approaches. First, by using the regression methods described above directly on the heritability estimated at each discovery step (i.e., with the accumulated number of loci found as new PMIDs are added). The second approach is a sampling-regression method that considers the change (i.e., regression) in the parameters of the distribution of effect sizes, as well as the change in the distribution of minor allele frequencies (MAF; the lowest of risk allele frequency, *q*, or 1 – *q*), in order to infer the expected heritability by sampling new candidate loci. For the distribution of effect sizes, the parametric distribution can be selected by the user, but a gaussian distribution is always assumed for MAF. Details of this process can be found in López-Cortegano and Caballero [9]. This second approach for inferring the heritability is more time demanding than the previous one, and could take several seconds to run if a high number of additional loci is entered, but it should never take more than one or two minutes of computation. Note that under this method it could occur that the predicted value of heritability decreases as more and more loci are assumed. This may appear as a contradiction, but it is not. It is just a consequence of the fact that loci effect sizes can be expected to decline severely as more and more loci are discovered. Thus, small effect sizes are becoming a majority in the expected distribution of effects and, therefore, the resampled loci may contribute less to heritability, even if they are more numerous.

## 3. Examples of Application

Here we will show different study cases to illustrate what kind of information can be extracted using GWEHS with regard to the architecture of complex traits and missing heritability, as well as its limitations. A complete description of the options used can be found in the README file, which can also be read in the ‘About’ section of the application. 

### 3.1. Exploring the Genetic Architecture

**Figure 1:**
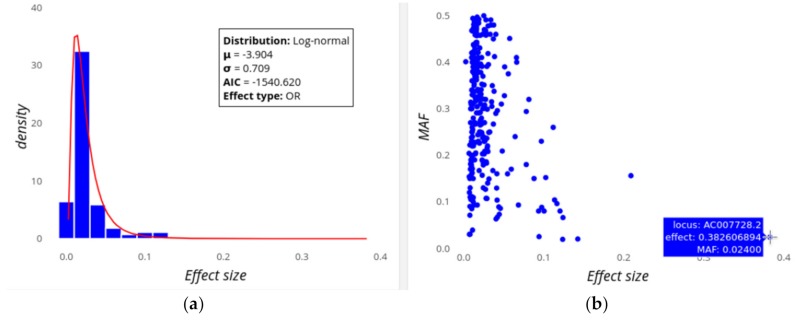
Screenshot of GWEHS (Genome-Wide Effect sizes and Heritability Screener) showing the distribution of loci effect sizes for Digestive disease (i.e., Crohn’s disease and Inflammatory bowel disease), using the default parameters. (**a**) The observed distribution of locus effect sizes (blue bars) and the fit to a log-normal distribution (red line), including a box with the distribution parameters, Akaike information criterion (AIC) value and the trait type. (**b**) Scatterplot illustrating the joint distribution of locus effect sizes and the minor allele frequency or MAF, (blue points). The interactive panel allows the user to explore the individual loci (SNPs) on the plot. In the figure, a blue box indicates the locus (gene) name, effect and MAF of the corresponding SNP with the highest effect size.

The first row of plots let the users explore the distribution of locus effect sizes and their contributions to heritability. Figure 1 shows the distribution of effect sizes for Digestive disease, which is a trait cluster composed by Crohn’s disease and Inflammatory bowel disease, assuming a prevalence of 0.32% (i.e., *k* = 0.0032). This cluster is based on their common genetic background, but note that we could also analyse them separately. On the left (Figure 1a) it can be seen that the best fit for the locus effect sizes for this trait is to a log-normal distribution with mean and standard deviation parameters µ = −3.90 and σ = 0.71. This is a leptokurtic distribution showing that low effect sizes are more common than high effect ones, but with a relatively scarce class closest to zero, perhaps because loci of very small effect are not usually found by GWAS. An alternative view of this distribution is that showing the effect size as well as the MAF for the individual loci (Figure 1b). This shows a tendency of higher effect sizes to have lower MAF values, while for lower effect sizes there is a much wider range of MAFs. Putting the cursor on any of the dots shows information on the particular locus (this will also work on bars, lines, etc.)

**Figure 2:**
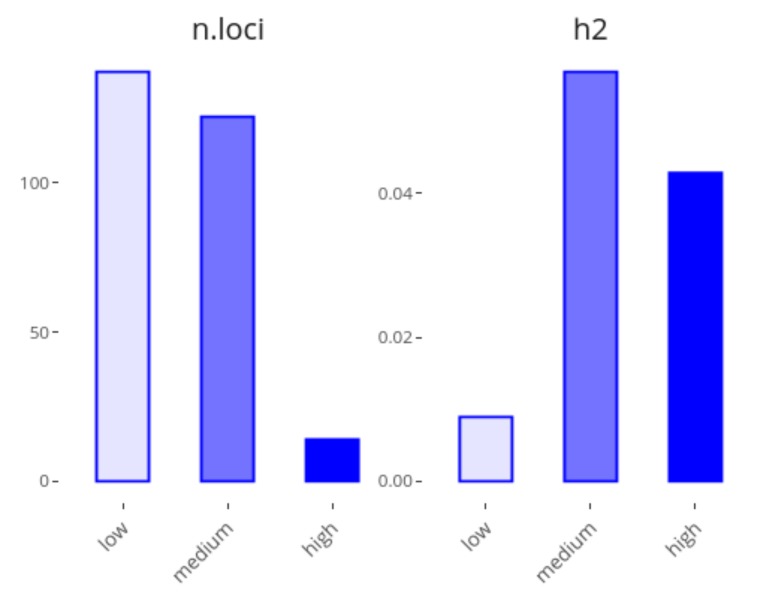
Screenshot of GWEHS (Genome-Wide Effect size and Heritability Screener) showing the locus contributions to heritability for Digestive disease (i.e., Crohn’s disease and Inflammatory bowel disease), using the default parameters, except for the percentage of low and high-effect size loci, which was set to 50% and 5%, respectively.

The second plot of the application shows the loci contributions to heritability by their effect size (Figure 2). To do so, loci are classified into three types of effect sizes: low, medium and high, each of these composed by 1/3 of all variants by default, though these values can be modified from 0 to 50% of contributing loci. For example, as can be seen in Figure 2, about 40% of the heritability of Digestive disease can be explained by the 5% of loci with the highest effects (14 loci), whereas a class including 50% of loci with the lowest effects (137 loci) explains only 8% of the heritability.

A third window on the right of the application shows a table including all selected loci (and their corresponding associated SNPs) for the previous analysis, including the original trait name they belong to, and their PMID, effect size, risk allele frequency and contribution to heritability (Table 1). This table can be conveniently sorted by one of these values, and downloaded to a file, allowing the users to use that filtered information for their own analyses. It also includes a box for searching text.

### 3.2. Inferring the Change in Mean Effect Size and Heritability

Regression analysis is used to fit the change of the mean effect size and heritability as new loci are being discovered with higher and higher sample sizes (or, alternatively, more recent PMIDs). By default, a two-parameter exponential regression is used, which usually gives the best fit according to previous results [9], though this method is not without problems, as it requires a starting value of its parameters to converge, and sometimes these cannot be found. For example, with the version of the Catalog used here, its fails to converge for the increase in heritability of Type 2 diabetes using the default parameters of GWEHS, though this can be solved by removing outliers of high effect size (this will remove for example locus AL05043.2, which is an extremely high effect, not replicated by any other study). Inference results cannot be obtained either for traits like Testicular germ cell tumor, because in the processed database only two observations remain with an accumulated different number of loci above 30, even when more than 3 PMIDs exist for this trait.

**Figure 3:**
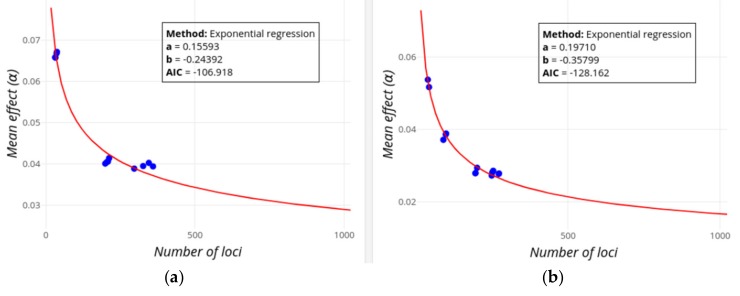
Screenshot of GWEHS (Genome-Wide Effect size and Heritability Screener) showing the inferences on the decrease of the mean effect size as new loci are found, using the default parameters (up to 1000 loci). Blue points indicate observed values from different sets of PubmedIDs (PMIDs). The red line corresponds to the regression fit. A text box includes the distribution parameters and the Akaike information criterion (AIC) value. (**a**) Inferences for Height. (**b**) Inferences for Digestive disease (Crohn’s disease and Inflammatory bowel disease).

**Figure 4:**
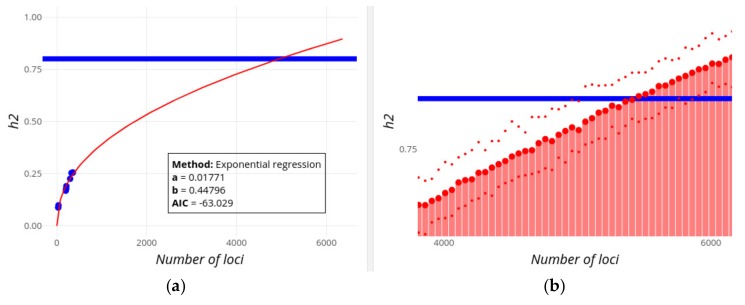
Screenshot of GWEHS (Genome-Wide Effect size and Heritability Screener) showing the inferences on the increase of heritability (*h*^2^) as new loci are found for human Height, using the default parameters (up to 6,000 loci). (**a**) Inferences using a regression method of the change in heritability with the number of loci. Blue points indicate observed values from different sets of PMIDs. The red line corresponds to the regression fit. A text box includes the distribution parameters and the Akaike information criterion (AIC) value. (**b**) Inferences using a regression method on the parameters of the distribution of effect sizes and minor allele frequencies (MAF), and sampling heritability values. The blue line indicates the assumed familial heritability. The red bars correspond to the heritability sampled. The red points show the median (large point), and 0.975 and 0.025 quantiles of that heritability (upper and lower small points, respectively). Note that this image has been zoomed, which is one of the interactive utilities supported by the R Shiny package.

**Figure 5:**
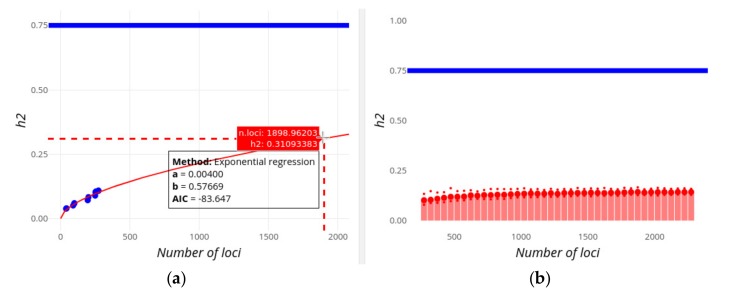
Screenshot of GWEHS (Genome-Wide Effect size and Heritability Screener) showing the inferences on the increase of heritability (*h*^2^) as new loci are found for Digestive disease (i.e., Crohn’s disease and Inflammatory bowel disease), using the default parameters. (**a**) Inferences using a regression method of the change in heritability with the number of loci. Blue points indicate observed values from different sets of PMIDs. The red line corresponds to the regression fit. Note that in this image we use the utility to toggle spike lines in order to read the expected value of *h*^2^ at a given number of loci (shown in a red box). A text box includes the distribution parameters and the Akaike information criterion (AIC) value. (**b**) Inferences using a regression method on the parameters of the distribution of effect sizes and MAF, and sampling heritability values. The blue line indicates the assumed familial heritability. The red bars correspond to the heritability sampled. The red points show the median (large point), and 0.975 and 0.025 quantiles of that heritability (upper and lower small points, respectively).

Figure 3, Figure 4 and Figure 5 below show the observed decrease in mean effect size and increase in heritability for two example traits (Height and Digestive disease) in which the GWAS-explained heritability is expected or not, respectively, to potentially reach the values of the familial heritability, assuming an exponential regression model. The values of the exponential regression parameters as well as the AIC values are shown in a box. When the pointer is put on any of the dots, information on the mean locus effect and the number of loci is provided together with the PMID of the study being added.

Figure 3 shows the decline in the mean effect size with an increasing number of loci. The figure shows a substantial decline of the mean effect after the initial loci are found, but the decline is only moderate as the population sample sizes progressively increase.

Figure 4a and Figure 5a show the increase in the explained heritability as more and more loci are being found with increasing population sample sizes. According to the exponential regression fit, the familial estimate of heritability of each of the traits (0.8 and 0.75, respectively; horizontal blue lines) could be potentially explained by more than 4,000 and 8,500 loci, respectively, always assuming an additive contribution of loci. If extremely high effect loci are removed with the ‘Remove outliers’ option, then the familial heritability of Digestive disease cannot ever be explained even if 10,000 additional candidate loci are considered. Note that the best fit for regressions on heritability (Figure 4a and Figure 5a) is to a linear regression for both traits, implying that the familial estimates of *h^2^* are reached by a number of loci lower than those observed in Figure 4 and Figure 5. It seems unlikely, however, that heritability will increase linearly with the number of loci, especially regarding the exponential or logistic decrease in the effect size of new found loci (in fact, it was exponential using previous releases of the GWAS Catalog), but this serves to illustrate that the inference of future contributions to heritability is not always straightforward. Some limitations of these methods will be discussed below.

An alternative, and more reliable, view on the missing number of loci is given in Figure 4b and Figure 5b, which correspond to the expected heritability sampled from the predicted distribution of loci effect sizes as new loci are being discovered. For Height (Figure 4b), the familial heritability can be explained with about 4,500 loci, in rough agreement with results from the alternative regression method (Figure 4a). For Digestive disease, however, this method suggests that the found heritability will reach an asymptote below *h^2^* = 0.25, and that the full familial heritability cannot apparently be explained adding the effects of more and more variants (even if 10,000 loci are considered).

### 3.3. Validation

To check the performance of the application, we re-evaluated some of the 16 traits analyzed by López-Cortegano and Caballero [9] using GWEHS, and found that the best fit for the loci effect sizes is again to a log-normal distribution (93.75% of traits), supporting our previous results even when a filtering based on a genomic distance between loci of 1 Mb is considered. In addition, most heritability is again explained by high or medium effect loci. It is to note, however, that the selection of traits has changed (e.g., the Neutrophil traits cluster is now represented by Neutrophil count and White blood cell count traits), because GWEHS does not cluster traits based on their genomic background at early pre-processing stages, but gives the choice of grouping traits later to the user.

The exponential regression is again the model that better explains the decrease in the mean effect size (40%) together with the logistic model (33%). As for the increase of the explained heritability, both the logistic and exponential regressions represent the best fit for a 40% of traits each. This balance turns in favor of the exponential regression if we only regard results where convergence to the model is achieved. We recommend using an exponential approach always when possible, as the logistic regression tends to give predictions that reach an upper limit of heritability immediately after the last observation (i.e., an exponential approach is more conservative). Using these models, about one third of the traits analyzed by López-Cortegano and Caballero [9] cannot have their heritability potentially explained by the addition of effects of new loci following the regression-sampling method, whereas that number decreases to three traits (Prostate cancer, Psoriasis and Body mass index) out of eleven, regarding the alternative regression method for those traits where the exponential model converged. If a logistic approach is assumed, results become less consistent among methods, as the regression-sampling method reaches the familial estimates of heritability for all traits, but the only-regression approach only does for two traits. The number of missing loci in the new analysis shows some differences with the previous inferences. For example, the analysis made here, incorporating new data, implies about 4,500 loci for Height, while the previous analysis suggested about 2,000 loci [9]. Overall, the results suggest again, that at least for some traits, the additive contribution of potentially missing loci cannot explain the observed familial heritability.

## 4. Discussion

We provide a new resource tool that will let researchers easily mine the GWAS Catalog and explore the genetic background and architecture of a large number of complex traits and diseases, as well as to make rough inferences on their missing heritability. This should give researchers the opportunity to check the state of knowledge of traits of interest, as well as to know how the different variants found by GWAS contribute to genetic variation for the trait or disease under study. This information is provided in an interactive and graphical environment, but can be conveniently downloaded to use in subsequent analyses. These analyses are mainly exploratory, however, and can be subject to some limitations (see below), therefore the results obtained should be taken with caution.

Up to date, the GWAS Catalog database contains results for more than 10^5^ SNP associations to more than 3,000 different publication studies, covering multiple and diverse complex traits and diseases, also in the number of hundreds. In this regard, GWEHS allows users to query updated results for a given number of human traits, including information on the most representative SNPs, the associated locus (i.e., the mapped gene name or inter-genic region), as well as the associated effect, risk allele frequency, contribution to heritability and significance level. These results can be conveniently downloaded in a spreadsheet format for subsequent analyses (see example data in Table 1). Other software packages have also been developed to query the GWAS Catalog, as ‘gwascat’ [19], and more recently ‘gwasrapidd’ [37], allowing to a more straightforward retrieval of information from the GWAS Catalog application interface [16], with minimum data processing. However, while these packages are focused on providing direct access to the GWAS Catalog data with integration to the R language, GWEHS is aimed not only to read the GWAS Catalog database in a user friendly and interactive way, but also to a posteriori analysis of variants effect size and their contribution to heritability. Because of this, GWEHS is also limited to a lower dataset of SNPs (i.e., it will not query the complete, raw GWAS Catalog), as only the most representative traits and results are considered for analyses. For example, GWEHS will only read information from traits with a polygenic and well-known genetic background of at least 30 different loci, as this is a requirement to fit their effects to a parametric distribution, meaning that diseases or traits influenced by a lower number of loci might by underrepresented. Furthermore, only highly significant variants (*P* ≤ 5·10^−8^) will be selected in order to avoid false positives, but also potentially reducing the number of selected variants.

The distribution of effect sizes has been a topic of extensive research, as it is fundamental to understand the genetic architecture of complex traits and diseases, e.g., if genetic variability and heritability are mainly explained by multiple low effect variants or a few variants of high effect sizes [2]. GWEHS allows different parametric distributions to be fitted, as well as estimating the corresponding distribution parameters (Figure 1a). It also enables extraction of the variants with extreme effect sizes from the table generated (e.g., as in Table 1), visualize them together with their MAF (Figure 1b) and/or remove them as outliers if desired. For example, the loci effect sizes for Digestive disease fit a log-normal distribution (Figure 1a), with locus AC007728.2 being that with the highest effect size (Figure 1b), although it is not that contributing most to heritability, as it is a rare variant. Regarding the loci contribution to heritability, a second plot responds to the question of whether low or high effect variants contribute most to heritability (Figure 2).

All analyses are of course limited to the accuracy of the corresponding estimates of effect sizes and frequencies. For example, analyses for OR traits are conditional to an estimate of prevalence, which is required to compute contributions to heritability. Although deviations from the true prevalence value have low impact for less prevalent diseases [33], we advise to use precise estimates, especially for inferential analyses (see below in this section). Furthermore, effects and frequencies could also be biased because population structure is not accounted for, and there could be admixture of different population ancestries (i.e., effect and frequencies are not corrected by ethnic group) and can contribute in fact as a source of missing heritability [38,39]. Unfortunately, this information is difficult to extract from the GWAS Catalog, as it is not always available, and when it is, it has text form, being more challenging to mine this information, especially when there is a lack of consensus on how to name different ethnicities, that could be hierarchized (e.g., it is not trivial to decide whether ‘Asian ancestry’ is the same or not in a particular study as ‘Chinese ancestry’ or ‘Han ancestry’). Similarly, other information as the particular model or covariates used in the individual GWAS performed is not included in the Catalog and cannot be used to correct effect size estimates. Finally, linkage disequilibrium (LD) could also affect results for some traits and loci, and it cannot be accounted for, since this is also a population-dependent parameter whose information is not available from the GWAS Catalog. As a result, the number of contributing loci, as well as the computed values of heritability, could be inflated if there are loci in LD, or if the same locus is named differently and thus classified as different loci by GWEHS. To avoid this possible bias, GWEHS includes the option to limit the number of loci in a window of a given size in Mega bases. This pruning method allows for alternative definitions of locus to be used (e.g., 1 Mb) [6,7], but it may imply a potential loss of information [40].

GWEHS provides a way to make inferences on the expected decrease of the mean locus effect size as well as the increase in the explained heritability (see Figure 3, Figure 4 and Figure 5), allowing a forecast of whether the current estimates of familial heritability could be explained by the additive contribution of loci yet to be found, and if so, the number of missing loci. For example, according to our results, it is unlikely that loci found by GWAS will ever explain the familial estimates of heritability for Digestive disease (Figure 5), while for Height the additive contribution of about 4,500 new loci could explain its familial estimate (Figure 4), even if these variants have lower and lower effect sizes (Figure 3a), though this does not mean it will be necessarily the case. The inference methods are, however, subject to some limitations, and caution is required when interpreting the results. For example, the exponential regression method could fail to converge. This could be fixed if several starting values of the regression parameters were tried, or if these could be provided by the user. In practice, however, this would greatly slow and complicate the performance of the application, without guarantee of success. Therefore, we decided to give the user the possibility to use alternative regression methods instead. In any case, being the application open-source, it gives researchers the opportunity to download the code and modify it at will for particular cases. On the other hand, the quality of the inferences will depend indeed on the quality (and quantity) of the data, so caution must be taken always when interpreting the results. For example, the number of data points could be low for some traits, compromising the quality of the regression analyses. In addition, the estimates of effect size could also be affected by winner’s curse, producing an upward bias of their estimates [41] and thus affecting the regression analyses. We tried to avoid this first by selecting only the most significant SNPs [42], and also by updating the effect size estimates of a given variant to that obtained with the largest population sample size (as explained in Methods) so that variants found with the lowest sample sizes (the most affected by the winner’s curse), have their effect and frequencies updated following studies with larger population sizes and statistic power, typically the most recent ones. Finally, inference results for heritability can be discordant between the only-regression method (Figure 4a and Figure 5a) and the one based on sampling-regression (Figure 4b and Figure 5b), usually with the first one predicting a value of heritability potentially explained by the addition of loci effects, and the latter reaching a lower asymptote. While the first regression method is simpler, with less underlying assumptions, the latter is based on more information (from effect sizes and MAF) and takes into account the expected distribution of effect sizes and the trait genetic architecture. Our recommendation is to consider both approaches, but give more credit to the second one even though it also has limitations.

## 5. Conclusions

A new software is provided to explore the GWAS Catalog and make inferences on the heritability of human traits and diseases. Despite the limitations, we expect this application to be of interest for the human genetics and genomics community, and that this tool will serve also to illustrate the communicating potential of tools based on simple markdown documents. Since these documents can be embed as chunks of executable code and graphs (e.g., R markdown) and interactive functions, they have the potential to become a strong standard to share results and to write scientific reports.

## Figures and Tables

**Table 1:** Illustration of the first rows in the table generated by GWEHS (Genome-Wide Effect size and Heritability Screener), showing example data for Digestive disease (i.e., Crohn’s disease and Inflammatory bowel disease), using the default parameters.

Trait	PMID	locus	SNP	effect	*q*	*h^2^*	−logP
Crohn’s disease	23128233	AC007728.2	rs2066847	0.383	0.024	0.007	208.222
Crohn’s disease	30500874	HLA-DQB1–MTCO3P1	rs184950714	0.209	0.156	0.011	18.398
Crohn’s disease	18587394	LINC02471	rs11175593	0.143	0.020	0.001	9.523
Crohn’s disease	22412388	LINC00491	rs7705924	0.124	0.066	0.002	7.699
Crohn’s disease	26192919	SLC2A13–LINC02555	rs12422544	0.124	0.019	0.001	24.398
Crohn’s disease	17554261	LINC01680–AL359081.1	rs10801047	0.120	0.080	0.002	7.523
Crohn’s disease	23850713	FCHSD2–AP002761.2	rs11235667	0.117	0.096	0.002	8.155
Crohn’s disease	22412388	AL645939.5	rs9258260	0.114	0.104	0.002	9.699
Crohn’s disease	21102463	NOD2	rs2076756	0.112	0.260	0.005	68.398

The table shows the SNPs sorted by decreasing effect size, including information on the trait name (Trait), publication (PMID), mapped gene or inter-genic region (locus), SNP identifier (SNP), effect size (effect), risk allele frequency (*q*), contribution to heritability (*h^2^*) and probability value (−logP).
